# Quantitative and Qualitative Phytochemical Analysis of *Manilkara zapota* (Sapodilla) Extract and Its Antibacterial Activity on Some Gram-Positive and Gram-Negative Bacteria

**DOI:** 10.1155/2023/5967638

**Published:** 2023-12-26

**Authors:** Shahla Hashemi Shahraki, Fereshteh Mohamadhasani Javar, Mehdi Rahimi

**Affiliations:** ^1^Biology Department, Faculty of Science, University of Sistan and Baluchestan, Zahedan, Iran; ^2^Department of Biology, Faculty of Science, Payame Noor University (PNU), Tehran, Iran; ^3^Department of Biotechnology, Institute of Science and High Technology and Environmental Sciences, Graduate University of Advanced Technology, Kerman, Iran

## Abstract

A molecule's antibacterial and antiviral action is exclusively linked to substances that selectively eradicate bacteria and viruses or inhibit their growth without significantly damaging adjacent tissues. The purpose of this research is to evaluate quantitative and qualitative phytochemical analysis and the antibacterial effects of *Manilkara zapota* fruit extract on some Gram-positive (*Staphylococcus aureus*, *Enterococcus faecalis*, *Micrococcus luteus*, *Bacillus cereus*, and *Listeria monocytogenes*) and Gram-negative (*Escherichia coli*, *Pseudomonas aeruginosa*, and *Klebsiella pneumoniae*) bacteria in laboratory conditions. Qualitative chemical screening was used to identify different classes of active chemical compounds, and quantitative analysis of the chemical composition of the plant was used to measure the contents of flavonoid, total phenol, anthocyanin, and antioxidant activity. Antibacterial effects of *Manilkara zapota* ethanol extract were determined by disk diffusion methods, minimum inhibitory concentration (MIC), and minimum bactericidal concentration (MBC). Qualitative chemical screening revealed the presence of flavonoids, tannins, quinones, terpenoids, and glycosides while the presence of saponins was not observed. The bacterial inhibition zones against *Listeria monocytogenes*, *Enterococcus faecalis*, *Pseudomonas aeruginosa*, *Staphylococcus aureus*, *Micrococcus luteus*, *Escherichia coli*, *Klebsiella pneumoniae*, and *Bacillus cereus* are 15.44 ± 0.33, 12.23 ± 0.11, 8.85 ± 0.2, 14.22 ± 0.33, 15 ± 0.44, 9.33 ± 0.13, 10.33 ± 0.36 and 14.55 ± 0.45 mm, respectively. MIC and MBC of the extract in Gram-positive bacteria were 25 and 50, and in Gram-negative bacteria were 50 and 100 mg/ml, respectively. The findings imply that *Manilkara zapota* extract includes a good amount of plant compounds and can be a significant source for a variety of uses, including antibacterial.

## 1. Introduction

In recent years, there has been a great interest in antibacterial herbal medicines, which can be attributed to the resistance of pathogens and the increasing popularity of traditional medicine [1]. On the other hand, excessive and unbalanced use of chemical compounds has led to resistance to bacteria and other microorganisms. Therefore, the effect of drugs has become weak and neutral and has led to an increase in the amount of drug consumption and a tendency to use compounds with newer and stronger formulations. Another problem with the use of chemical drugs is the increase in their side effects, which lead to diseases that can be more dangerous than the original disease [2]. Plants are considered sources of various chemical substances, effective substitutes for synthetic antibacterial agents with minimal side effects [3]. Plants are a source of potential and useful chemicals such as phenols, phenolic derivatives (quinones, flavones, flavonoids, flavonols, tannins, coumarins, etc.), terpenoids, essential oils, alkaloids, lectin, and polypeptides [4]. Indeed, plants use a vast and largely unknown reservoir of substances to defend against microorganisms, insects, and herbivores. According to the approach for the use of medicines and herbal products, the study of the medicinal properties of plants is of particular importance.

Evidence to support the therapeutic claims of plants against various diseases can be discovered by identifying and evaluating plants and their plant compounds. Advanced methods like high-performance liquid chromatography (HPLC) [5], gas chromatography (GC) [6], and thin layer chromatography (TLC) [7] are very useful for both qualitative and quantitative detection of phytoconstituents. However, when these techniques are unavailable or inaccessible, conventional phytochemical tests that are cost-effective, simple, and require few resources are often a good alternative for initial phytochemical screening. Based on the precipitation reaction, foamy appearance, and color change, the qualitative analysis of the chemical compounds of the plant is done [7].

The *Manilkara zapota* plant belonging to the Sapotaceae family has many medicinal effects. Different parts of the *Manilkara zapota* tree are used in traditional medicine [8]. The ability to purge the digestive system as well as diuretic and tonic is the characteristic of the seeds [9]. The tree's bark has antidiarrheal, astringent, and antibiotic effects. The fruit is also used for antidiarrhea and to treat pulmonary diseases. The leaves are used to treat cough, cold, and diarrhea. Its seeds and leaves show significant antibacterial activity. Abdel Monem et al. reported that *Manilkara zapota* leaves have antihyperglycemic and hypocholesterolemic activities. They also showed that aqueous and alcoholic leaf extracts have antioxidant activity [10].

Since medicinal plants are natural sources of chemicals, in this research, the antibacterial effects of *Manilkara zapota* extract on some bacteria (*Listeria monocytogenes*, *Enterococcus faecalis*, *Pseudomonas aeruginosa*, *Staphylococcus aureus*, *Micrococcus luteus*, *Escherichia coli*, *Klebsiella pneumoniae*, and *Bacillus cereus*) in laboratory conditions were investigated.

## 2. Materials and Methods

### 2.1. Experimental Research

This study complied with relevant institutional, national, and international guidelines and legislation of Iran. In this research, *Manilkara zapota* fruit was collected from Chabahar Gardens in Zahedan City. To prepare the extract, the fruits were first dried in the shade and then powdered. The resulting powder was extracted by the maceration method. During this process, 100 g of the powder was mixed with an organic solvent of 80% ethanol and placed in a shaker at a temperature of 25°C for 24 hour. To separate the large parts, the content was passed through the Whatman No. 1 filter paper. After these steps, the concentrated solution was placed in a 40°C incubator to obtain a dry powder from the extract. The obtained powder was stored in a dark glass container in the refrigerator until use. The obtained powder was used for quantitative and qualitative phytochemical analysis and antibacterial activity.

### 2.2. Quantitative and Qualitative Phytochemical Analysis

Qualitative chemical screening was used to identify different classes of active chemical compounds such as saponins, flavonoids, tannins, quinones, terpenoids, and glycosides [11]. To detect saponins, 1 ml of extract was added to 1 ml of distilled water and shaken vigorously. The formation of foam confirms the presence of saponin. To detect flavonoids, a few drops of dilute sodium hydroxide (NaOH) solution were added to 1 ml of the extract, whereby an intense yellow color appeared. If the solution becomes colorless after adding a few drops of dilute hydrochloric acid (HCl), this is a sign of the presence of flavonoids. For tannin, 1 ml of extract was mixed with 5% FeCl_3_. The color of the black-green sediment confirms the presence of tannins. To reveal the presence of glycoside, 1 ml of the extract was added to sulfuric acid (H_2_SO_4_) and glacial acetic acid (2 ml) containing a few drops of iron chloride. A purple ring may form below the brown ring, indicating the presence of a glycoside. To test quinones, 0.5 ml of concentrated hydrochloride was added to 1 ml of extract. The presence of quinones is indicated by the production of a yellow precipitate. To identify terpenoids, 1 ml of extract was mixed with 5 ml of concentrated H_2_SO_4_ and 2 ml of chloroform in a mixture. Reddish-brown color indicates the presence of terpenoids. Quantitative analysis of the chemical composition of the *Manilkara zapota* fruit was used to measure the content of flavonoid, total phenol, anthocyanin, and antioxidant activity. The basis of quantitative photometric measurement of the absorption process is according to the Beer–Lambert law. UV-visible spectroscopy can be used to estimate the flavonoid content. About 0.025 g of powder is ground with ethyl alcohol : acetic acid (99 : 1, v : v). After centrifugation and placing in a water bath at 80°C, the absorbance of the sample was read in a UV-visible spectrophotometer at wavelengths of 270, 300, and 330 nm [12]. To determine the anthocyanin content, 0.001 g of fruit powder was ground with 10 ml of acidic methanol and placed in the dark for 24 hour. After centrifugation, the absorbance was read at a wavelength of 550 nm [13]. Antioxidant potential was evaluated by the DPPH (2,2-diphenyl-1-picrylhydrazyl) method by Ishaque et al. The absorbance was measured at 517 nm to evaluate the amount of residual DPPH [14]. Total phenol content was estimated by the Folin-reagent method. The absorbance was measured at a wavelength of 725 nm by using a UV-vis spectrophotometer [15].

### 2.3. Antibacterial Activity

Bacterial strains of *Listeria monocytogenes*, *Enterococcus faecalis*, *Pseudomonas aeruginosa*, *Staphylococcus aureus*, *Micrococcus luteus*, *Escherichia coli*, *Klebsiella pneumoniae*, and *Bacillus cereus* were obtained from the Kerman University of Medical Sciences. The reference culture of the mentioned microorganisms was kept in the refrigerator, and the culture was renewed every month in nutrient agar. To validate the antibacterial effect of *Manilkara zapota* plant extract, the methods of diffusion in agar with the help of disk (disc diffusion), minimum inhibitory concentration (MIC), and minimum bactericidal concentration (MBC) were used. At first, 200 mg of powder was dissolved in 1 ml of ethanol (80%) to prepare the extract solution. In the solution of the ethanolic extract of the tested plant, several blank disks with a diameter of 6 mm were inserted and remained in the solution for one hour. By sterile tweezers and under aseptic conditions, the discs were removed from the extract and transferred to a sterile plate to dry completely [16]. Discs soaked in consumable solvent were considered as test controls. To perform the disc diffusion test, one loop of the reference culture of each bacterium was transferred to the medium of nutrient broth and incubated for 24 hour. Then, the resulting antibacterial suspension was diluted to reach a turbidity equal to half McFarland. In the next step, with the help of a sterile swap, a sample was taken from this suspension and cultured in Mueller–Hinton agar medium. Finally, with the help of sterile tweezers, discs were placed on the medium at a distance of 15 mm from the edge of the plate and the plate was incubated for 24 hour at 37°C. The solvent was considered as negative control and chloramphenicol antibiotic as the positive control. The diameter of the inhibition zone of the extract was measured by using a millimeter ruler [17]. The minimum inhibitory concentration (MIC) and minimum bactericidal concentration (MBC) were determined by the broth dilution method. At first, different dilutions of the studied fruit extracts were prepared. The mentioned dilutions included 10 dilutions of 0.5 to 200 mg/ml, which were prepared by the sequential dilution method and from the stock solution of the extracts. To determine MIC, 1 ml of 24 hour bacterial suspension grown in nutrient broth and brought to half McFarland turbidity was added to each dilution. The tubes were incubated for 24 hours at a temperature of 37°C. After this period, the lowest concentration in which bacterial growth was inhibited was selected as MIC. To calculate the minimum bactericidal concentration (MBC) of the extract from the MIC test treatment tubes, 100 *μ*l was placed on the Mueller–Hinton agar medium without the extract and spread on the surface of the plate with the help of a sterile glass spreader. The plate with the lowest concentration of infected extract in which bacteria did not grow after a 24 hour incubation period at 37°C was determined as MBC [18, 19]. The data obtained from this study were statistically analyzed using SPSS software and one-way ANOVA statistical test.

## 3. Results

Preliminary phytochemical screening of various bioactive compounds (tannins, saponins, flavonoids, quinones, terpenoids, and glycosides) was performed in the *Manilkara zapota* extract. Among them, the presence of tannins, flavonoids, quinones, terpenoids, and glycosides was observed, while the presence of saponins was not observed (Table 1).

Table 2 shows the amount of total phenol, anthocyanin, flavonoid content, and antioxidant activity of *Manilkara zapota* fruit extract. The data show that the amounts of total phenol, anthocyanin, flavonoid 270, 300, 330 content, and antioxidant activity of *Manilkara zapota* extract were 0/54 ± 0.02, 40 ± 0.98, 5 ± 0.03, 94 ± 0.5, 34 ± 0.44, and 59 ± 0.13, respectively.

The results of the effect of *Manilkara zapota* extract on some bacteria (*Listeria monocytogenes*, *Enterococcus faecalis*, *Pseudomonas aeruginosa*, *Staphylococcus aureus*, *Micrococcus luteus*, *Escherichia coli*, *Klebsiella pneumoniae*, and *Bacillus cereus*) are shown in Table 3. The results show that *Manilkara zapota* extract prevented the growth of all tested bacteria. This inhibitory effect on inhibiting the growth of *Staphylococcus aureus*, *Micrococcus luteus*, *Listeria monocytogenes*, and *Bacillus cereus* bacteria was the highest. The least inhibitory effect of *Manilkara zapota* extract was on *Klebsiella pneumoniae*, *Pseudomonas aeruginosa*, and *Escherichia coli*.

The results of the minimum inhibitory concentration (MIC) and minimum bactericidal concentration (MBC) of *Manilkara zapota* extract are given in Table 4. These results show that the concentration of 25 mg/ml of the extract has an inhibitory effect on the bacteria *Staphylococcus aureus*, *Enterococcus faecalis*, *Micrococcus luteus*, *Listeria monocytogenes*, and *Bacillus cereus*, while the concentration of 50 mg/ml of the extract has an inhibitory effect on the bacteria *Pseudomonas aeruginosa*, *Klebsiella pneumonia*, and *Escherichia coli*.

The results of minimum bactericidal concentration (MBC) of *Manilkara zapota* fruit extract on *Staphylococcus aureus*, *Enterococcus faecalis*, *Micrococcus luteus*, *Klebsiella pneumoniae*, *Bacillus cereus*, *Listeria monocytogenes*, and *Pseudomonas aeruginosa* were 50, 50, 50, 100, 50, 50, and 100 mg/ml respectively. These results indicate that among the tested bacteria, there was the least sensitivity in the case of *Klebsiella pneumoniae*, *Pseudomonas aeruginosa*, and *Escherichia coli*.

## 4. Discussion

In this study, it was found that the extract of *Manilkara zapota* fruit showed a good antibacterial effect. Various chemical compounds present in medicinal plants can lead to the antibacterial activity of plant extracts. Phytochemical analysis of *Manilkara zapota* extract showed the presence of tannins, flavonoids, quinones, terpenoids, and glycoside compounds. The antibacterial activity of *Manilkara zapota* fruit extract may be due to the presence of the above plant chemicals. Tannins and terpenoids are plant metabolites known for their antimicrobial activity [20].

Similar to our results, Thiago et al. reported a complete inhibition of the growth of *Staphylococcus aureus*, *Pseudomonas aeruginosa*, and *Escherichia coli* caused by the aqueous extract of *Manilkara zapota* leaves [21]. Bhargavi et al. reported that methanolic and aqueous extracts of *Manilkara zapota* roots showed antibacterial activity against *Staphylococcus aureus* and *Escherichia coli* [22].

Acetone and methanol extracts of *Manilkara zapota* seeds were active against Gram-positive and Gram-negative organisms. The MIC values of potent extracts against sensitive organisms were between 53 and 380 *μ*g/ml [23]. In this study, concentrations of about 12.5 mg/ml of *Manilkara zapota* fruit extract prevent the growth of Gram-positive bacteria, while a higher concentration is needed to affect Gram-negative bacteria. Therefore, Gram-positive bacteria are more sensitive than Gram-negative bacteria. This difference in the response of Gram-negative and Gram-positive bacteria can be related to the difference in the structure of the outer membrane [24]. Gram-positive bacteria do not have an outer membrane compared to Gram-negative bacteria. The corresponding outer membrane acts as a barrier with limited permeability that prevents the penetration of some antibiotic components and drugs into the cell [25]. The presence of polysaccharides in the outer membrane prevents the passage of large and hydrophobic molecules. Since most of the effective compounds in the extracts and essential oils are hydrophobic, it can be concluded that these substances cannot penetrate and access the active points inside Gram-negative bacteria. For this reason, Gram-negative bacteria usually show more resistance to plant compounds compared to Gram-positive bacteria. Bangar et al. reported that chemical compounds in *Manilkara zapota*, such as anthocyanins, alkaloids, flavonoids, polyphenolic compounds, tannins, and triterpenoids, play an important role in antibacterial activity [26]. Tannins are water-soluble chemical compounds that are widely found in the plant kingdom and exhibit strong antibacterial properties. Various mechanisms that exist in tannins to inhibit antibacterial growth include deprivation of iron through iron chelation, disruption of metabolic activities of bacteria through inhibition of oxidative phosphorylation, and deprivation of essential compounds for bacterial growth. Polyphenol compounds also have strong antibacterial activity [27]. Inhibition of RNA and DNA, cytoplasmic membrane depolarization, and inhibition of macromolecular synthesis are some of the underlying antibacterial mechanisms of polyphenol compounds. Therefore, it seems that the compounds in *Manilkara zapota* fruit cause the antibacterial property of *Manilkara zapota*. In general, according to the above, the authors suggest that *Manilkara zapota* fruit extract can be used in the pharmaceutical industry as well as in the food industry as a biological preservative. More research is needed to detail their mechanism of action.

## 5. Conclusions

*Manilkara zapota* fruits were once widely consumed, but today, not much attention is paid to them. Therefore, it is urgently necessary to raise public awareness of this plant's therapeutic potential so that this fruit can be used to combat microbial strains that are extremely susceptible. While saponins were not found, preliminary phytochemical screening of a variety of bioactive chemicals revealed the presence of tannins, flavonoids, quinones, terpenoids, and glycosides. The findings imply that the *Manilkara zapota* extract includes a good amount of plant compounds and can be a significant source for a variety of uses, including antibacterial.

## Figures and Tables

**Table 1:** Qualitative phytochemical composition of *Manilkara zapota*.

Phytochemicals	+/−
Saponins	−
Flavonoids	+
Tannins	+
Quinones	+
Terpenoids	+
Glycosides	+

+ indicates presence; − indicates absence.

**Table 2:** Quantitative analysis of total phenol, anthocyanin, flavonoid contents, and antioxidant activity in extracts of *Manilkara zapota*

Parameter	Amount
Total phenol contents (mg/g GA)	0.54 ± 0.02
Anthocyanin contents (mmol/g DW)	40 ± 0.98
Flavonoid contents 270 (%)	5 ± 0.03
Flavonoid contents 300 (%)	94 ± 0.5
Flavonoid contents 330 (%)	34 ± 0.44
Antioxidant activity (%)	59 ± 0.13

**Table 3:** Size of inhibition zone diameter of *Manilkara zapota* extracts against some bacteria.

Bacteria	Size of inhibition zone diameter (mm)	Control (−)	Control (+)
*Escherichia coli*	9.33 ± 0.13	5 ± 0.53	33 ± 0.55
*Staphylococcus aureus*	14.22 ± 0.33	−	36 ± 0.71
*Enterococcus faecalis*	12.23 ± 0.11	6 ± 0.03	36 ± 0.88
*Micrococcus luteus*	15 ± 0.44	6 ± 0.66	21 ± 0.32
*Klebsiella pneumoniae*	10.33 ± 0.36	−	23 ± 0.44
*Bacillus cereus*	14.55 ± 0.45	5 ± 0.45	26 ± 0.55
*Listeria monocytogenes*	15.44 ± 0.33	−	26 ± 0.33
*Pseudomonas aeruginosa*	8.85 ± 0.2	5 ± 0.23	26 ± 0.23

− indicates the absence of antimicrobial activity in the extract.

**Table 4:** Minimum inhibitory concentration and minimum bactericidal concentration of *Manilkara zapota* extracts on bacteria.

Bacteria	Minimum inhibitory concentration (MIC) (mg/ml)	Minimum bactericidal concentration (MBC) (mg/ml)
*Escherichia coli*	50	100
*Staphylococcus aureus*	25	50
*Enterococcus faecalis*	25	50
*Micrococcus luteus*	25	50
*Klebsiella pneumoniae*	50	100
*Bacillus cereus*	25	50
*Listeria monocytogenes*	25	50
*Pseudomonas aeruginosa*	50	100

## Data Availability

All relevant data used to support the findings of this study are included within the article.
